# Supplementary material for: Heat adaptation of phage T7 under an extended genetic code
Source: Virus Evol. 2021 Dec 1;7(2):veab100. doi: 10.1093/ve/veab100 (PMC8923235; doi:10.1093/ve/veab100)
Supplement: veab100_Supp [file veab100_supp.zip › Sequencing_Analysis.zip!/Sequencing_Analysis/S1/evidence/SNP_11.html]

BRESEQ :: Evidence


| Predicted mutation | | | | | | |
| --- | --- | --- | --- | --- | --- | --- |
| evidence | seq id | position | mutation | annotation | gene | description |
| RA | NC\_001604.1 | 29,258 | A→G | intergenic (–/–) | – / – | –/– |

| Read alignment evidence... | | | | | | | | | | | |
| --- | --- | --- | --- | --- | --- | --- | --- | --- | --- | --- | --- |
|  | seq id | position | | ref | new | freq | score (cons/poly) | reads | annotation | genes | product |
| \* | NC\_001604.1 | 29,258 | 0 | A | G | 99.5% | 11525.7 / NA | 3789 | intergenic (–/–) | *–/–* | –/– |
| Reads supporting (aligned to +/- strand):  **ref** base A (6/3);  **major** base G (2044/1711);  **minor** base T (13/6);  **total** (2066/1723) | | | | | | | | | | | |
| Fisher's exact test for biased strand distribution *p*-value = 2.55e-01 | | | | | | | | | | | |
| Kolmogorov-Smirnov test that lower quality scores support variant *p*-value = 1.00e+00 | | | | | | | | | | | |

Only 100 of 3800 total aligned reads displayed.  

`GCGTCGTCCAGACTCTGCTGACTTCTTTGAGAAGTATATCGACAACGGTCTGGTTACTGGCGCAATCCCATCTGATGCTCAAGCCACACAGCTTATAAGCCAAGCGTTCAGTGACGCTTCTAGCCGTGCTGGTGGTGCTGACTTCCTGATGCGAGTCGGTGACAAGAAGGTAACACTTAACGGAGCCACTACGACTTACCGAGAGTTGATTGGTGAGGAACAGTGGAACGCTCTCATGGTCACAGCACAACGTTCTCAGTTTGAGACTGACGCGAAGCTGAACGAGCAGTATCGCTTGAAGATTAACTCTGCGCTGAACCAAGAGGACCCAAGGACAGCTTGGGAGATGCTTCAAGGTATCAAGGCTGAACTAGATAAGGTCCAACCTGATGAGCAGATGACACCACAACGTGAGTGGCTAATCTCCGCACAGGAACAAGTTCAGAATCAGATGAACGCATGGACGAAAGCTCAGGCCAAGGCTCTGGACGATTCCATGAAGTCAATGAACAAACTTGACGTAATCGACAAGCAATTCCAGAAGCGAATCAACGGTGAGTGGGTCTCAACGGATTTTAAGGATATGCCAGTCAACG  >  NC_001604.1/28960‑29555`  

                                                                                                                                                                                                                                                                                                          `|`                                                                                                                                                                                                                                                                                                           
   `tcgtcCAGACTCTGCTGACTTCTTTGAGAAGTATATCGACAACGGTCTGGTTACTGGCGCAATCCCATCTGATGCTCAAGCCACACAGCTTATAAGCCAAGCGTTCAGTGACGCTTCTAGCCGTGCTGGTGGTGCTGACTTCCTGATGCGAGTCGGTGACAAGAAGGTAACACTTAACGGAGCCACTACGACTTACCGAGAGTTGATTGGTGAGGAACAGTGGAACGCTCTCATGGTCACAGCACAACGTTCTCAGTTTGAGACTGACGCGAAGCTGAACGAGCAGTATCGCTTGGAGAtt                                                                                                                                                                                                                                                                                                      >  1:491646/1‑301 (MQ=255)`  
     `gtcCAGACTCTGCTGACTTCTTTGAGAAGTATATCGACAACGGTCTGGTTACTGGCGCAATCCCATCTGATGCTCAAGCCACACAGCTTATAAGCCAAGCGTTCAGTGACGCTTCTAGCCGTGCTGGTGGTGCTGACTTCCTGATGCGAGTCGGTGACAAGAAGGTAACACTTAACGGAGCCACTACGACTTACCGAGAGTTGATTGGTGAGGAACAGTGGAACGCTCTCATGGTCACAGCACAACGTTCTCAGTTTGAGACTGACGCGAAGCCGAACGAGCAGTATCGCTTGGAGATTaa                                                                                                                                                                                                                                                                                                    >  1:82010/1‑301 (MQ=255)`  
      `agatgtgtataagagacagtgtGAGAAGTATATCGACAACGGTCTGGTTACTGGCGCAATCCCAGCTGATGCTCAAGCCACACAGCTTATAAGCCAAGCGTTCAGTGACGCTTCTAGCCGTGCTGGTGGTGCTGACTTCCTGATGCGAGTCGGTGACAAGAAGGTAACACTTAACGGAGCCACTACGACTTACCGAGAGTTGATTGGTGAGGAACAGTGGAACGCTCTCATGGTCACAGCACAACGTTCTCAGTTTGAGACTGACGCGAAGCTGAACGAGCAGTATCGCTTGGAGATTAAc                                                                                                                                                                                                                                                                                                   <  1:166404/280‑1 (MQ=255)`  
         `aGACTCTGCTGACTTCTTTGAGAAGTATATCGACAACGGTCTGGTTACTGGCGCAATCCCATCTGATGCTCAAGCCACACAGCTTATAAGCCAAGCGTTCAGTGACGCTTCTAGCCGTGCTGGTGGTGCTGACTTCCTGATGCGAGTCGGTGACAAGAAGGTAACACTTAACGGAGCCACTACGACTTACCAAGAGTTGATTGGTGAGGAACAGTGGAACGCTCTCATGGTCACAGCACAACGTTCTCAGTTTGAGACTGACGCGAAGCTGAACGAGCAGTATCGCTTGGAGATTAActct                                                                                                                                                                                                                                                                                                >  1:254531/1‑301 (MQ=255)`  
            `ctctGCTGACTTCTTTGAGAAGTATATCGACAACGGTCTGGTTACTGGCGCAATTCCATCTGATGCTCAAGCCACACAGCTTATAAGCCAAGCGTTCAGTGACGCTTCTAGCCGTGCTGGTGTTGCTGACTTCCTGATGCGAGTCGGTGACAAGAAGGTAACACTTAACGGAGCAACTACGACTTACCGAGAGTGGATTGGTGAGGAAAAGTGGAACGCACTAATGGTCACAGCACAACGTGCTCAGTTTGAACCTGACACGAAGATGAACAAGAAGTATCGCCTGGGGATTAACTATgcg                                                                                                                                                                                                                                                                                             >  1:117429/1‑301 (MQ=255)`  
             `caggctgACTTCTTTGAGAAGTATATCGACAACGGTCTGGTTACTGGCGCAATCCCATCTGATGCTCAAGCCACACAGCTTATAAGCCAAGCGTTCAGTGACGCTTCTAGCCGTGCTGGTGGTGCTGACTTCCTGATGCGAGTCGGTGACAAGAAGGTAACACTTAACGGAGCCACTACGACTTACCGAGAGTTGATTGGTGAGGAACAGTGGAACGCTCTCATGGTCACAGCACAACGTTCTCAGATTGAGACTGAAGCGAAGCTGAACGAGAAGTATCGCTTGGAGAATAACACcgcgg                                                                                                                                                                                                                                                                                            >  1:505095/4‑300 (MQ=255)`  
                  `tgACTTCTTTGAGAAGTATATCGACAACGGTCTGGTTACTGGCGCAATCCCATCTGATGCTCAAGCCACACAGCTTATAAGCCAAGCGTTCAGTGACGCTTCTAGCCGTGCTGGTGGTGCTGACTTCCTGATGCGAGTCGGTGACAAGAAGGTAACACTTAACGGAGCCACTACGACTTACCGAGAGTTGATTGGTGAGGAACAGTGGAACGCTCTCATGGTCACAGCACAACGTTCTCAGTTTGAGACTGACGCGAAGCTGAACGAGCAGTATCGCTTGGAGATTAACTCTGCGCTGAAc                                                                                                                                                                                                                                                                                       <  1:594520/301‑1 (MQ=255)`  
                   `gACTTCTTTGAGAAGTATATCGACAACGGTCTGGTTACTGGCGCAATCCCATCTGATGCTCAAGCCACACAGCTTATAAGCCAAGCGTTCAGTGACGCTTCTAGCCGTGCTGGTGGTGCTGACTTCCTGATGCGAGTCGGTGACAAGAAGGTAACACTTAACGGAGCCACTACGACTTACCGAGCGTTGATTGGTGAGGAACAGTGGAACGCTCTCATGGTCACAGCACAACGTTCTCAGTTTGAGACTGACGCGAAGCTGAACGAGCAGTATCGCTTGGAGATTAACTCTGCGCTGAAcc                                                                                                                                                                                                                                                                                      >  1:408203/1‑301 (MQ=255)`  
                   `gACTTCTTTGAGAAGTATATCGACAACGGTCTGGTTACTGGCGCAATCCCATCTGATGCTCAAGCCACACAGCTTATAAGCCAAGCGTTCAGTGACGCTTCTAGCCGTGCTGGTGGTGCTGACTTCCTGATGCGAGTCGGTGACAAGAAGGTAACACTTAACGGAGCCACTACGACTTACCGAGAGTTGATTGGTGAGGAACAGTGGAACGCTCTCATGGTCACAGCACAACGTTCTCAGTTTGAGACTGACGCGAAGCTGAACGAGCAGTATCGCTTGGAGATTAActcctgtctcttat                                                                                                                                                                                                                                                                                      >  1:649862/1‑290 (MQ=255)`  
                     `cttcttTGAGAAGTATATCGACAACGGTCTGGTTACTGGCGCAATCCCATCTGATGCTCAAGCCACACAGCTTATAAGCCAAGCGTTCAGTGACGCTTCTAGCCGTGCTGGTGGTGCTGACTTCCTGATGCGAGTCGGTGACAAGAAGGTAACACTTAACGGAGCCACTACGACTTACCGATAGTTGATTGGTGAGGAACAGTGGAACGCTCTCATGGTCACAGCACAACGTTCTCAGTTTGAGACTGACGCGAAGCTGAACGAGCAGTATCGCTTGGAGATTAACCCTGCGCCTAACCaa                                                                                                                                                                                                                                                                                    >  1:87968/1‑301 (MQ=255)`  
                              `gaAGTATATCGACAACGGTCTGGTTACTGGCGCAATCCCATCTGATGCTCAAGCCACACAGCTTATAAGCCAAGCGTTCAGTGACGCTTCTAGCCGTGCTGGTGGTGCTGACTTCCTGATGCGAGTCGGTGACAAGAAGGTAACACTTAACGGAGCCACTACGACTTACCGAGAGTTGATTGGTGAGGAACAGTGGAACGCTCTCATGGTCACAGCATAACGTTCTCAGTTTGAGACTGACGCGAAGCCGAACGAGCAGTATCGCTTGGAGATTAACTCTGCGCTGAACCAAGAGGACCCa                                                                                                                                                                                                                                                                           >  1:134516/1‑301 (MQ=255)`  
                              `gaAGTATATCGACAACGGTCTGGTTACTGGCGCAATCCCATCTGATGCTCAAGCCACACAGCTTATAAGCCAAGCGTTCAGTGACGCTTCTAGCCGTGCTGGTGGTGCTGACTTCCTGATGCGAGTCGGTGACAAGAAGGTAACACTTAACGGAGCCACTACGACTTACCGAGAGTTGATTGGTGAGGAACAGTGGAACGCTCTCATGGTCACAGCACAACGTTCTCAGTTTGAGACTGACGCGAAGCTGAACGAGCAGTATCGCTTGGAGATTAACTCTGCGCTTAACCAAGAGGACCCa                                                                                                                                                                                                                                                                           >  1:93405/1‑301 (MQ=255)`  
                                 `gTATATCGACAACGGTCTGGTTACTGGCGCAATCCCATCTGATGCTCAAGCCACACAGCTTATAAGCCAAGCGTTCAGTGACGCTTCTAGCCGTGCTGGTGGTGCTGACTTCCTGATGCGAGTCGGTGACAAGAAGGTAACACTTAACGGAGCCACTACGACTTACCGAGAGTTGATTGGTTAGGAACAGTGGAACGCTCTCATGGTCACAGCACAACGTTCTCAGTTTGAGACTGACGCGAAGCTGAACGAGCAGTATCGCTTGGAGATTAAcctgtctcttatacacatctccgagccc                                                                                                                                                                                                                                                                        >  1:395519/1‑274 (MQ=255)`  
                                 `gTATATCGACAACGGTCTGGTTACTGGCGCAATCCCATCTGATGCTCAAGCCACACAGCTTATAAGCCAAGCGTTCAGTGACGCTTCTAGCCGTGCTGGTGGTGCTGACTTCCTGATGCGAGTCGGTGACAAGAAGGTAACACTTAACGGAGCCACTACGACTTACCGAGAGTTGATTGGTGAGGAACAGTGGAACGCTCTCATGGTCACAGCACAACGTTCTCAGTTTGAGACTGACGCGAAGCTGAACGAGCAGTATCGCTTGGAGATTAACTCTGCGCTGAACCAAGAGGAcccctgt                                                                                                                                                                                                                                                                        >  1:100092/1‑297 (MQ=255)`  
                                 `gTATATCGACAACGGTCTGGTTACTGGCGCAATCCCATCTGATGCTCAAGCCACACAGCTTATAAGCCAAGCGTTCAGTGACGCTTCTAGCCGTGCTGGTGGTGCTGACTTCCTGATGCGAGTCGGTGACAAGAAGGTAACACTTAACGGAGCCACTACGACTTACCGAGAGTTGATTGGTGAGGAACAGTGGAACGCTCTCATGGTCACAGCACAACGTTCTCAGTTTGAGACTGACGCGAAGCTGAACGAGCAGTATCGCTTGGAGATTAACTCTGCGCTGAACCAAGAGGACCCAAgg                                                                                                                                                                                                                                                                        >  1:662476/1‑301 (MQ=255)`  
                                 `gTATATCGACAACGGTCTGGTTACTGGCGCAATCCCATCTGATGCTCAAGCCACACAGCTTATAAGCCAAGCGTTCAGTGACGCTTCTAGCCGTGCTGGTGGTGCTGACTTCCTGATGCGAGTCGGTGACAAGAAGGTAACACTTAACGGAGCCACTACGACTTACCGAGAGTTGATTGGTGAGGAACAGTGGAACGCTCTCATGGTCACAGCACAACGTTCTCAGTTTGAGACTGACGCGAAGCTGAACGAGCAGTATCGCTTGGAGATTAACTCTGCGCTGAACCAAGAGGACCCAAgg                                                                                                                                                                                                                                                                        >  1:513728/1‑301 (MQ=255)`  
                                   `atatCGACAACGTTCTGGTTACTGGCACAATCCCATCTGATGCTTAAGCCACACAGCTTATAAGCCAAGCGTTCAGTGACGCTTCTAGCCGTGCTGGTGGTGCTGACTTCCTGATGCGAGTCGGTGACAAGAAGGTAACACTTAACGGAGCCACTACGACTTACCGAGAGTTGATTGGTGAGGAACAGTGGAACGCTCTCATGGTCACAGCACAACGTTCTCAGTTTGAGACTGACGCGAAGCTGAACGAGCAGTATCGCTTGGAGATTAACTCTGCGCTGAACCAAGAGGACCCAAGGAc                                                                                                                                                                                                                                                                      <  1:190264/301‑1 (MQ=255)`  
                                   `agatCGACAACGGTCTGGTTACTGGCGCAATCCAATCTGATGCTCAAGCCACACAGCTTATAAGCCAAGCGTTAAGTGACGCTTCTAGCCGTGCTGGTGGTGCTGACTTCCTGATGCGAGTCGGTGACAAGAAGGTAACACTTAACGGAGCCACTACGACTTACCGAGAGTTGATTGGTGAGGAACAGTGGAACGCTCTCATGGTCACAGCACAACGTTCTCAGTTTGAGACTGACGCGAAGCTGAACGAGCAGTATCGCTTGGAGATTAACTCTGCGCTGAACCAAGAGGACCCAAGGAc                                                                                                                                                                                                                                                                      <  1:383049/299‑1 (MQ=255)`  
                                           `aaCGGTCTGGTTACTGGCGCAATCCCATCTGATGCTCAAGCCACACAGCTTATAAGCCAAGCGTTCAGTGACGCTTCTAGCCGTGCTGGTGGTGCTGACTTCCTGATGCGAGTCGGTGACAAGAAGGTAACACTTAACGGAGCCACTACGACTTACCGAGAGTGGATTGGTGAGGAACAGTGGAACGCTCTCATGGTCACAGCACAACGTTCTCAGTTTGAGACTGACGCGAAGCTGAACGAGCAGTATCGCTTGGAGATTAACTCTGCGCTGAACCAAGAGGACCCAAGGACAGCTTggg                                                                                                                                                                                                                                                              <  1:292032/301‑1 (MQ=255)`  
                                             `gggTCTGGTTACTGGCGCAATCCCATCTGATGCTCAAGCCACACAGCTTATAAGCCAAGCGTTCAGTGACGCTTCTAGCCGTGCTGGTGGTGCTGACTTCCTGATGCGAGTCGGTGACAAGAAGGTAACACTTAACGGAGCCACTACGACTTACCGAGAGTTGATTGGTGAGGAACAGTGGAACGCTCTCATGGTCACAGCACAACGTTCTCAGTTTGAGACTGACGCGAAGCTGAACGAGCAGTATCGCTTGGAGATTAACTCTGCGCTGAACCAAGAGGACCCAAGGACAGCTTGGgag                                                                                                                                                                                                                                                            <  1:333142/300‑1 (MQ=255)`  
                                             `gggTCTGGGTACTGGCGGAATACCATCTGAGGCTCAAGCCACACAGCTTATAAGCCAAGCGTTAAGTGACGCTTATAGCCGTGCTGGTGGTGCTGACTTCCGGATGCGAGTCGGTGACAAGAAGGTAACACTTAACGGAGCCACTACGACTTACCGAGAGTTGATTGGTGAGGAACAGTGGAACGCTCTCATGGTCACAGCACAACGTTCTCAGTTTGAGACTGACGCGAAGCTGAACGAGCAGTATCGCTTGGAGATTAACTCTGCGCTGATCCAAGAGGACCCAAGGACAGCTTGGgag                                                                                                                                                                                                                                                            <  1:562109/300‑1 (MQ=255)`  
                                             `cGGTTTGGTGACTGGCGCAATCCCGTCTGATGCTCAAGCCACACAGCTTATAAGCCAAGCGTTCAGTGACTTTTCTAGCCGTGCTGGTGGTGCTGACTTCCTGATGCGAGTCGGTGACAAGAAGGTAACACTTAACGGAGCCACTACGACTTACCGAGAGTTGATTGGTGAGGAACAGTGGAACGCTCTCATGGTCACAGCACAACGTTCTCAGTTTGAGACTGACGCGAAGCTGAACGAGCAGTATCGCTTGGAGATTAACTCTGCGCTGAACCAAGAGGACCCAAGGACAGCTTGGgag                                                                                                                                                                                                                                                            <  1:172794/301‑1 (MQ=255)`  
                                             `cGGTCTGGTTACTGGCGCAATACCATCTGATGCTCAAGCCACACAGCTTATAAGCCAAGCGTTCAGTGACGCTTCTAGCCGTGCTGGTGGTGCTGACTTCCTGATGCGAGTCGGTGACAAGAAGGTAACACTTAACGGAGCCACTACGACTTACCGAGAGTTGATTGGTGAGGAACAGTGGAACGCTCTCATGGTCACAGCACAACGTTCTCAGTTTGAGACTGACGCGAAGCTGAACGAGCAGTATCGCTTGGAGATTAACTCTGCGCTGAACCAAGAGGACCCAAGGACAGCTTGGgag                                                                                                                                                                                                                                                            >  1:568006/1‑301 (MQ=255)`  
                                              `ggTCTGTTTACTGGCGCAATCCCATCCGACGCTCAAGCCACACAGCTTATAAGCCAAGCGTTCAGTGACGCTTCTAGCCGTGCTGGTGGTGCTGACTTCCTGATGCGAGTCGGTGACAAGAAGGTAACACTTAACGGAGCCACTACGACTTACCGAGAGTTGATTGGTGAGGAACAGTGGAACGCTCTCATGGTCACAGCACAACGTTCTCAGTTTGAGACTGACGCGAAGCTGAACGAGCAGTATCGCTTGGAGATTAACTCTGCGCTGAACCAAGAGGACCCAAGGACAGCTTGGgaga                                                                                                                                                                                                                                                           <  1:439876/301‑1 (MQ=255)`  
                                                   `ggTTACTGGCGCAATCCCATCTGATGCTCAAGCCACACAGCTTATAAGCCAAGCGTTCAGTGACGCTTCTAGCCGTGCTGGTGGTGCTGACTTCCTGATGCGAGTCGGTGACAAGAAGGTAACACTTAACGGAGCCACTACGACTTACCGAGAGTTGATTGGTGAGGAACAGTGGAACGCTCTCATGGTCACAGCACAACGTTCTCAGTTTGAGACTGACGCGAAGCTGAACGAGCAGTATCGCTTGGATATTAACTCTGCGCTGAACCAAGAGGACCCAAGGCCAGCTTGGGAGATGCtt                                                                                                                                                                                                                                                      >  1:622299/1‑301 (MQ=255)`  
                                                   `ggTTACTGGCGCAATCCCATCTGATGCTCAAGCCACACAGCTTATAAGCCAAGCGTTCAGTGACGCTTCTAGCCGTGCTGGTGGTGCTGACTTCCTGATGCGAGTCGGTGACAAGAAGGTAACACTTAACGGAGCCACTACGACTTACCGAGAGTTGATTGGTGAGGAACAGTGGAACGCTCTCATGGTCACAGCACAACGTTCTCAGTTTGAGACTGACGCGAAGCTGAACGAGCAGTATCGCTTGGAGATTAACTCTGCGCTGAACCAAGAGGACCCAAGGACAGCTTGGGAGATGCtt                                                                                                                                                                                                                                                      >  1:601094/1‑301 (MQ=255)`  
                                                       `aCTGGCGCAATCCCATCTGATGCTCAAGCCACACAGCTTATAAGCCAAGCGTTCAGTGACGCTTCTAGCCGTGCTGGTGGTGCTGACTTCCTGATGCGAGTCGGTGACAAGAAGGTAACACTTAACGGAGCCACTACGACTTACCGAGAGTTGATTGGTGAGGAACAGTGGAACGCTCTCATGGTCACAGCACAACGTTCTCAGTTTGAGACTGACGCGAAGCTGAACGAGCAGTATCGCTTGGAGATTAACTCTGCGCTGAACCAAGAGGACCCAAGGACAGCTTGGGAGATGCTTCAAg                                                                                                                                                                                                                                                  <  1:454608/301‑1 (MQ=255)`  
                                                        `cgggCGCAATCCCATCTGATGCTCAAGCCACACAGCTTATAAGCCAAGCGTTCAGTGACGCTTCTAGCCGTGCTGGTGGTGCTGACTTCCTGATGCGAGTCGGGGACAAGAAGGTAACACTTAACGGAGCCACTACGACTTACCGAGAGTTGATGGGTGAGGAACAGTGGAAGGCTCTCATGGTCACAGCACAACGTTCTCAGTTTGAGACTGAGGCGAAGCTGAACGAGCAGTAGCGCTTGGAGATTAACTCTGCGCTGAACCAAGAGGACCCAAGGACAGCTTGGGAGATGCTTCAAgg                                                                                                                                                                                                                                                 <  1:366053/299‑1 (MQ=255)`  
                                                        `cTGGCGCAATCCCATCTGATGCTCAAGCCACACAGCTTATAAGCCAAGCGTTCAGTGACGCTTCTAGCCGTGCTGGTGGTGCTGACTTCCTGATGCGAGTCGGTGAAAAGAAGGTAACACTTAACGGAGCCACTACGACTTACCGAGAGTTGATTGGTGAGGAACAGTGGAACGCTCTCATGGTCACAGCACAACGTTCTCAGTTTGAGACTGACGCGAAGCTGAACGAGCAGTATCGCTTGGAGATTAACTCTGCGCTGAACCAAGAGGACCCAAGGACAGCTTGGGAGATGCTTCAAgg                                                                                                                                                                                                                                                 <  1:228939/301‑1 (MQ=255)`  
                                                          `tgcgcAATCCCATCTGATGCTCAAGCCACACAGCTTATAAGCCAAGCGTTCAGTGACGCTTCTCGCCGTGCTCTTTCTGCTGACTTCCTTTTGCGAGTCTTTTACAAGAAGGTCCCACTTAACGGAGCCACTTCGACTTACCTCTTGTTGATTGGTCTGTCACCGTGGAACGCTCTCATGGTCACAGCTCAACGTTCTCAGTTTGAGTCTGACTCGAAGCTGAACGTGTAGTATCGCTTGGAGATTAACTCTGCGCTGAACCAAGAGGACCCAAGTACACCTTGGGAGCTGTTTctttttt                                                                                                                                                                                                                                               >  1:106175/2‑295 (MQ=255)`  
                                                           `gcgcAATCCCATCTGATGCTCAAGCCACACAGCTTATAAGCCAAGCGTTCAGTGACGCTTCTAGCCGTGCTGGTTGTGCTGACTTCCTGATGCGAGTCGGTGACAAGAAGGTAACACTTAACGGAGCCACTACGACTTACCGAGAGTTGATTGGTGAGGAACAGTGGAACGCTCTCATGGTCACATCACAACGTTCTCAGTTTGAGACTGACGCGAAGCTGAACGAGCAGTATCGCTTGGAGATTAACTCTGCGCTGAACCGAGAGGACCCAAGGACAGCTTGGGAGATGCTTCAAGGTAt                                                                                                                                                                                                                                              >  1:128747/1‑301 (MQ=255)`  
                                                            `cgcAATCCCATCTGATGCTCAAGCCACACAGCTTATAAGCCAAGCGTTCAGTGACGCTTCTAGCCGTGCTGGTGGTGCTGACTTCCTGATGCGAGTCGGTGACAAGAAGGTAACACTTAACGGAGCCACTACGACTTACCGAGAGTTGATTGGTGAGGAACAGTGGAACGCTCTCATGGTCACAGCACAACGTTCTCAGTTTGAGACTGACGCGAAGCTGAACGAGCAGTATCGCTTGGAGATTAACTCTGCGCTGAACCAAGAGGACCCAAGGACAGCTTGGGAGATGCTTCAAGGTATc                                                                                                                                                                                                                                             >  1:475204/1‑301 (MQ=255)`  
                                                               `aaTCCCATCTGATGCTCAAGCCACACAGCTTATAAGCCAAGCGTTCAGTGACGCTTCTAGCCGTGCTGGTGGTGCTGACTTCCTGATGCGAGTCGGTGACAAGAAGGTAACACTTAACGGAGCCACTACGACTTACCGAGAGTTGATTGGTGAGGAACAGTGGAACGCTCTCATGGTCACAGCACAACGTTCTCAGTTTGAGACTGACGCGAAGCTGAACGAGCAGTATCGCTTGGAGATTAACTCTGCGCTGAACCAAGAGGACCCAAGGACAGCTTGGGAGATGCTTCAAGGTATCAAg                                                                                                                                                                                                                                          <  1:551784/301‑1 (MQ=255)`  
                                                                `aTCCCATCTGATGCTCAAGCCACACAGCTTATAAGCCAAGCGTTCAGTGACGCTTCTAGCCGTGCTGGTGGTGCTGACTTCCTGATGCGAGTCGGTGACAAGAAGGTAACACTTAACGGAGCCACTACGACTTACCGAGAGTTGATTGGTGAGGAACAGTGGAACGCTCTCATGGTCACAGCACAACGTTCTCAGTTTGAGACTGACGCGAAGGTGAACGAGCAGTATCGCTTGGAGATTAACTCTGCGCTGAACCAAGAGGACCCAAGGACAGCGTGGGAGATGCTTCAAGGTATCAAgg                                                                                                                                                                                                                                         >  1:575964/1‑301 (MQ=255)`  
                                                                  `cccATCTGATGCTCAAGCCACACAGCTTATAAGCCAAGCGTTCAGTGACGCTTCTAGCCGTGCTGGTGGTGCTGACTTCCTGATGCGAGTCGGTGACAAGAAGGTAACATTTAACGGTGCCACTACGACTTACCGAGAGTTGATTGGTGAGGAACAGTGGAACGCTCTCATGGTCACAGCACAACGTTCTCAGTTTGAGACTGACGCGAAGCTGAACGAGCAGTATCGCTTGGAGATTAACTCTGCGCTGAACCAAGAGGACCCAAGGACAGCTTGGGAGATCCTTCAAggctgtatgtta                                                                                                                                                                                                                                       >  1:61734/1‑291 (MQ=255)`  
                                                                     `aTCTGATGCTCAAGCCACACAGCTTATAAGCCAAGCGTTCAGTGACGCTTCTAGCCGTGCTGGTGGTGCTGACTTCCTGATGCGAGTCGGTGACAAGAAGGTAACACTTAACGGAGCCACTACGACTTACCGAGAGTTGATTGGTGAGGAACAGTGGAACGCTCTCATGGTCACAGCACAACGTTCTCAGTTTGAGACTGACGCGAAGCTGAACGAGCAGTATCGCTTGGAGATTAACTCTGCGCTGAACCAAGAGGACCCAAGGACAGCTTGGGAGATGCTTCAAGGTATCAAGGCTGaa                                                                                                                                                                                                                                    >  1:53590/1‑301 (MQ=255)`  
                                                                      `tCTGATTCTCAAGCCACACAGCTTATAAGCCAAGCGTTCAGTGACGCTTCTAGCCGTGCTGGTGGTGCTGACTTCCTGATGCGAGTCGGTGACAAGAAGGTAACACTTAACGGAGCCACTACGACTTACCGAGAGTTGATTGGTGAGGAACAGTGGAACGCTCTCATGGTCACAGCACAACGTTCTCAGTTTGAGACTGACGCGTAGCTGAACGAGCAGTATCGCTTGGAGATTAACTCTGCGCTGAACCAAGAGGACCCAAGGACAGCTTGGGAGATGCTTCAAGGTATCAAGGCTGAAc                                                                                                                                                                                                                                   >  1:344477/1‑301 (MQ=255)`  
                                                                      `tCTGATGCTCAAGCCACACGGCTTATAAGCCAAGCGTTCAGTGACGCTTCTAGCCGTGCTGGTGGTGCTGACTTCCGGATGCGAGGCGGTGACAAGAAGGTAACACTTAACGGAGCCACTACGACTTACCGAGAGTTGATTGGTGAGGAACAGTGGAACGCTCTCATGGTCACAGCACAACGTTCTCAGTTTGAGACTGACGCGAAGCTGAACGAGCAGTATCGCTTGGAGATTAACTCTGCGCTGAACCAAGAGGACCCAAGGACAGCTTGGGAGATGCTTCAAGGTATCAAGGCTGAAc                                                                                                                                                                                                                                   <  1:183274/301‑1 (MQ=255)`  
                                                                           `tGCTCAAGCCACACAGCTTATAAGCCAAGCGTTCAGTGACGCTTCTAGCCGTGCTGGTGGTGCTGACGTCCTGATGCGAGTCGGTGACAAGAAGGTAACACTTAACGGAGCCACTACGACTTACCGAGAGTTGATTGGTGAGGAACAGTGGAACGCTCTCATGGTCACAGCACAACGTTCTCAGTTTGAGACTGACGCGAAGCTGAACGAGCAGTATCGCTTGGAGATTAACTCTGCGCTGAACCAAGAGGACCCAAGGACAGCTTGGGAGATGCTTCAAGGTATCAAGGCTGAACTAGAt                                                                                                                                                                                                                              <  1:198059/301‑1 (MQ=255)`  
                                                                           `ggCTCAAGCCACACAGCTTATAAGCCAAGCGTTCAGTGACGCTTCTAGCCGTGCTGGTGGTGCTGACTTCCTGATGCGAGTCGGTGACAAGAAGGTAACACTTAACGGAGCCACTACGACTTACCGAGAGTTGATTGGTGAGGAACAGTGGAACGCTCTCATGGTCACAGCACAACGTTCTCAGTTTGAGACTGACGCGAAGCTGAACGAGCAGTATCGCTTGGAGATTAACTCTGCGCTGAACCAAGAGGACCCAAGGACAGCTTGGGAGATGCTTCAAGGTATCAAGGCTGAACTAGAt                                                                                                                                                                                                                              <  1:435166/300‑1 (MQ=255)`  
                                                                            `gCTCAAGCCCCACAGCTGATAAGCCAAGCGTTCAGTGACGCTTCTAGCCGTGCTGGTGGTGCTGACTTCCTGATGCGAGTCGGTGACAAGAAGGTAACACTTAACGGAGCCACTACGACTTACCGAGAGTTGATTGGTGAGGAACAGTGGAACGCTCTCATGGTCACAGCACAACGTTCTCAGTTTGAGACTGACGCGAAGCTGAACGAGCAGTATCGCTTGGAGATTAACTCTGCGCTGAACCAAGAGGACCCAAGGACAGCTTGGGAGATGCTTCAAGGTATCAAGGCTGAACTAGATa                                                                                                                                                                                                                             <  1:525145/301‑1 (MQ=255)`  
                                                                               `cAAGCCACACAGCTTATAAGCCAAGCGTTCAGTGACGCTTCTAGCCGTGCTGGTGGTGCTGACTTCCTGATGCGAGTCGGTGACAAGAAGGTAACACTTAACGGAGCCACTACGACTTACCGAGAGTTGATTGGTGAGGAACAGTGGAACGCTCTCATGGTCACAGCACAACGTTCTCAGTTTGAGACTGACGCGAAGCTGAACGAGCAGTATCGCTTGGAGATTAACTCTGCGCTGAACCAAGAGGACCACAGGAAAGCTTCGAAGATGCTTCAAGGTATCAAGGCTGAACTAGATactg                                                                                                                                                                                                                          >  1:627662/1‑298 (MQ=255)`  
                                                                                `gccagacacaGCTTATAAGCCAAGCGTTCAGTGACGCTTCTAGCCGTGCTGGTGGTGCTGACTTCCTGATGCGAGTCGGTGACAAGAAGGTAACACTTAACGGAGCCACTGCGACTTACCGAGAGTTGATTGGTGAGGAACAGTGGAACGCTCTCATGGTCACAGCACAACGTTCTCAGTTTGAGACTGACGCGAAGCAGAACGAGCAGTATCGCTTGGAGATTAACTCTGCGCTGAACCAAGAGGACCCAAGGACAGCTTGGGAGATGCTTCAAGGTATCAAGGCTGAACTAGATAAGGt                                                                                                                                                                                                                         <  1:260419/296‑1 (MQ=255)`  
                                                                                `aagcccacaaGCTTATAAGACAAGCGTTAAGTGACGGTTCTAGCCGTGCTGGTGGGGCTGACGTCCTGATGCGAGTCGGTGACAAGAAGGTAACACTTAACGGAGCCACTACGACTTACCGAGAGTTGATTGGTGAGGAACAGTGGAACGCTCTCATGGTCACAGCACAACGTTCTCAGTTTGAGACTGACGCGAAGCTGAACGAGCAGTATCGCTTGGAGATTAACTCTGCGCTGAACCAAGAGGACCCAAGGACAGCTTGGGAGATGCTTCAAGGTATCAAGGCTGAACTAGATAAGGt                                                                                                                                                                                                                         <  1:426030/292‑1 (MQ=255)`  
                                                                                `aaGCCACACAGCTTATAAGCCAAGCGTTCAGTGACGCTTCTAGCCGTGCTGGTGGTCCTGACTTCCTGATGCGAGTCGGTGCCAAGAAGGTAACACTTAACGGAACCACTACGACTTACCGAGAGTTGATTGGTGAGGAACAGTGGAACGCTCTCATGGCCGCAGCACAACGTTCTCAGTTTGAGACTGACGCGAAGCTGAACGAGCAGTATCGCTTGGAGATTACCGCTGCGCTGAACCAAGAGGACCCAAGGACAGCTTGGGAGAGGCTTCAAGGGACCAAGGCTGAACTAGGTAAGGt                                                                                                                                                                                                                         >  1:240917/1‑301 (MQ=255)`  
                                                                                     `gacaggCTTATAAGCCAAGCGTTCAGTGACGCTTCTAGCCGTGCTGGTGGTGCTGACTTCCTGATGCGAGTCGGTGACAAGAAGGTAACACTTAACGGAGCCACTACGACTTACCGAGAGTTGATAGGTGAGGAACAGTGGAACGCTCTCATGGTCACAGCACAACGTTCTCAGTTTGAGACTGACGCGAAGCTGAACGAGCAGTATCGCTTGGAGATTAACTCTGCGCTGAACCAAGAGGACCCAAGGACAGCTTGGGAGATGCTTCAAGGTATCAAGGCTGAACTAGATAAGGTCCAAc                                                                                                                                                                                                                    <  1:247785/296‑1 (MQ=255)`  
                                                                                      `cacaGTTAATAAGCCAAGCGTTCAGTGACGCTTCTAGCCGTGCTGGTGGTGCTGACTTCCTGATGCGAGTCGGTGACAAGAAGGTAACACTTAACGGAGCCACTACGACTTACCGAGAGTTGATTGGTGAGGAACAGTGGAACGCTCTCATGGTCACAGCACAACGTTCTCAGTTTGAGACTGACGCGAAGCTGAACGAGCAGTATCGCTTGGAGATTAACTCTGCGCTGAACCAAGAGGACCCAAGGACAGCTTGGGAGATGCTTCAAGGTATCAAGGCTGAACTAGATAAGGTCAAAcc                                                                                                                                                                                                                   <  1:480691/301‑1 (MQ=255)`  
                                                                                      `cacaGCTTATAAGCCAAGCGTTCAGTGACGCTTCTAGCCGTGCTGGTGGTGCTGACGTCCTGATGCGAGTCGGTGACAAGAAGGTAACACTTAACGGAGCCACTACGACTTACCGAGAGTTGATTGGTGAGGAACAGTAGAACGCTCTCATGGTCACAGCACAACGTTCTCAGTTTGAGACTGACGCGAAGCTGAACGAGCAGTATCGCTTGGAGATTAACTCTGCGCTGAACCAAGAGGACCCAAGGACAGCTTGGGAGATGCTTCAAGGTATCAAGGCTGAACTAGATAAGGTCCAAcc                                                                                                                                                                                                                   <  1:63923/301‑1 (MQ=255)`  
                                                                                          `gCTTATAAGCCAAGCGTTCAGTGACGCTTCTAGCCGTGCTGGTGGTGCTGACTTCCTGATGCGAGTCGGTGACAAGAAGGTAACACTTAACGGAGCCACTACGACTTACCGAGAGTTGATTGGTGAGGAACAGTGGAACGCTCTCATGGTCACAGCACAACGTTCTCAGTTTGAGACTGACGCGAAGCTGAACGAGCAGTATCGCTTGGAGATTAACTCTGCGCTGAACCAAGAGGACCCAAGGACAGCTTGGGAGATGCTTCAAGGTATCAAGGCTGAACTAGATAAGGTctgtctcttt                                                                                                                                                                                                               >  1:208202/1‑292 (MQ=255)`  
                                                                                          `gCTTATAAGCCAAGCGTTCAGTGACGCTTCTAGCCATGCTGGTGGTTCTGACTTCCTGATGCGAGTCGGTGACAAGAAGGTAACACTTAACGGAGCCACTACGACTTACCGAGAGTTGATTGGTGAGGAACAGTGGAACGCTCTCATGGTCACAGCACAACGTTCTGAGTTTGAGACTGACGCGAAGCTGAACGAGCAGTATCGCTTGGAGATTAACTCTGCGCTGAACCAAGAGGACCCAAGGACAGCTTGGGAGATGCTTCAAGGTATCAAGGATGAACTAGATAAGGTCCCACCtgag                                                                                                                                                                                                               >  1:530162/1‑300 (MQ=255)`  
                                                                                           `gttATAAGCCAAGCGTTCAGTGACGCTTCGAGCCGTGCTGGTGGTGCTGACTTCCTGATGCGAGTCGGTGACAAGAAGGTAACACTTAACGGAGCCACTACGACTTACCGAGAGTTGATTGGTGAGGAACAGTGGAACGCTCTCATGGTCACAGCACAACGTTCTCAGGTTGAGACTGACGCGAAGCTGAACGAGCAGTATCGCTTGGAGATTAACTCTGCGCTGAACCAAGAGGACCCAAGGACAGCTTGGGAGATGCTTCAAGGTATCAAGGCTGAACTAGATAAGGTCCAACCtgatg                                                                                                                                                                                                              <  1:351323/300‑1 (MQ=255)`  
                                                                                           `cTTATAAGCCAAGCGTTCAGTGACGCTTCTAGCCGTGCTGGTGGTGCCGACTTCCTGATGCGAGTCGGTGACAAGAAGGTAACACTTAACGGAGCCACTACGACTTACCGAGAGTTGATTGGTGAGGAACAGTGGAACGCTCTCATGGTCACAGCACAACGTTCTCAGTTTGAGACTGACGCGAAGCTGAACGAGCAGTATCGCTTGGAGATTAACTCTGCGCTGAACCAAGAGGACCCAAGGACAGCTTGGGAGATGCTTCAAGGTATCAAgctgtctcttatacacatctccgagccca                                                                                                                                                                                                              >  1:460581/1‑273 (MQ=255)`  
                                                                                              `aagagacaggCGGTCAGTGACGCTTCGAGCCGTGCTGGTGGTGCTGACTTCCTGATGCGAGTCGGTGACAAGAAGGTAACACTTAACGGAGCCACTACGACTTACCGAGAGTTGATTGGTGAGGAACAGTGGAACGCTCTCATGGTCACAGCACAACGTTCTCAGTTTGAGACTGACGCGAAGCTGAACGAGCAGTATCGCTTGGAGATTAACTCTGCGCTGAACCAAGAGGACCCAAGGACAGCTTGGGAGATGCTTCAAGGTATCAAGGCTGAACTAGATAAGGTCCAACCTGATGAGc                                                                                                                                                                                                           <  1:358391/292‑1 (MQ=255)`  
                                                                                                 `aGCCAATCGTTCAGTGACGCTTCTAGCCTTGCTGGTGGTGCTGACGTCCTGATGCGAGTTGGTGACAAGAAGGTAACACTTAACGGAGCCACTACGACTTACCGAGAGTTGATTGGTGAGGAACAGTGGAACGCTCTCATGGTCACAGCACAACGTTCTCAGTTTGAGACTGACGCGAAGCTGAACGAGCAGTATCGCTTGGAGATTAACTCTGCGCTGAACCAAGAGGACCCAAGTACAGCGTGGGAGATGCTTCAAGGTATCAAGGCTGAACTAGATAAGGTCCAACCTGATGAGCAGa                                                                                                                                                                                                        <  1:122909/301‑1 (MQ=255)`  
                                                                                                 `aGCCAAGCGTTCAGTGACGCTTCTAGCCGTGCTGGTGGTGCTGACTTCCTGATGCGAGTCGGTGACAAGAAGGTAACACTTAACGGAGCCACTACGACTTACCGAGAGTTGATTGGTGAGGAACAGTGGAACGCTCTCATGGTCACAGCACAACGTTCTCAGTTTGAGACTGACGCGAAGCTGAACGAGCAGTATCGCTTGGAGATTAACTCTGCGCTGAACCAAGAGGACCCAAGGACAGCTTGGGAGATGCTTCAAGGTATCAAGGCTGAACTAGATAAGGTCCAACCTGATGAGCAGa                                                                                                                                                                                                        >  1:267205/1‑301 (MQ=255)`  
                                                                                                  `gCCAAGCGTTCAGTGACGCTTCTAGCCGTGCTGGTGGTGCTGACTTCCTGATGCGAGTCGGTGACAAGAAGGTAACACTTAACGGAGCCACTACGACTTACCGAGAGTTGATTGGTGAGGAACAGTGGAACGCTCTCATGGTCACAGCACAACGTTCTCAGTTTGAGACTGACGCGAAGCTGAACGAGCAGTATCGCTTGGAGATTAACTCTGCGCTGAACCAAGAGGACCCAAGGACAGCTTGGGAGATGCTTCAAGGTATCAAGGCTGAACTAGATAAGGTCCAACCTGATGAGcctgt                                                                                                                                                                                                       >  1:389138/1‑297 (MQ=255)`  
                                                                                                   `ccAAGCGTTCAGTGACGCTTCTAGCCGTGCTGGTGGTGCTGACTTCCTGATGCGAGTCGGTGACAAGAAGGTAACACTTAACGGAGCCACTACGACTTACCGAGAGTTGATTGGTGAGGAACAGTGGAACGCTCTCATGGTCACAGCACAAAGTTCTCAGTTTGAGACTGACGCGAAGCTGAACGAGCAGTATCGCTTGGAGATTAACTCTGCGCTGAACCAAGAGGACCCAAGGACAGCTTGGGAGATGCTTCAAGGTATCAAGGCTGAACTAGATAAGGTCCAACCTGATGAGCAGATg                                                                                                                                                                                                      <  1:315462/301‑1 (MQ=255)`  
                                                                                                       `gCGTTCAGTGACGCTTCTAGCCGTGCTGGTGGTGCTGACTTCCTGATGCGAGTCGGTGACAAGAAGGTAACACTTAACGGAGCCACTACGACTTACCGAGAGTTGATTGGTGAGGAACAGTGGAACGCTCTCATGGTCACAGCACAACGTTCTCAGTTTGAGACTGACGCGAAGCTGAACGAGCAGTATCGCTTGGAGATTAACTCTGCGCTGAACCAAGAGGACCCAAGGACAGCTTGGGAGATGCTTCAAGGTATCAAGGCTGAACTAGATAAGGTccctgtctcttatacacatctcc                                                                                                                                                                                                  >  1:588147/1‑280 (MQ=255)`  
                                                                                                        `cGTTCAGTAAGGCTTCTAGTCGTGCTGGTGGGGCTGACTTCCTGATGCGAGTCGGTGACAAGAAGGTAACACTTAACGGAGCCACTACGACTTACCGAGAGTTGATTGGTGAGAAACAGTGGAACGCTCTCATGGTCACAGCACAACGTTCTCAGTTTGAGACTGACGCGAAGCTGAACGGGCAGTATCGCTTGGAGATTAACTCTGCGCTGAACCAAGAGGACCCAAGGACAGCTTGGGAGATGCTTCAAGGTATCAAGGCTGAACTAGATAAGGTCCAACCTGATGAGCAGATGAcacc                                                                                                                                                                                                 <  1:498728/301‑1 (MQ=255)`  
                                                                                                          `ttCAGTGACGCTTCTAGCCGTGCTGGTGGTGCTGACTTCCGGATGCGAGTCGGTGACAAGAAGGTAACACTTAACGTAGCCACTACGACTTACCGAGAGTTGATTGGTGAGGAACAGTGGAACGCTCTCATGGTCACAGCACAACGTTCTCAGTTTGAGACTGACGCGAAGCTGAACGAGCAGTATCGCTTGGAGATTAACTCTGCGCTGAACCAAGAGGACCCAAGGACAGCTTGGGAGATGCTTCAAGGTATCAAGGCTGAACTAGATAAGGTCCAACCTGATGAGCAGATGAcaccac                                                                                                                                                                                               <  1:70448/301‑1 (MQ=255)`  
                                                                                                              `gacatgCTTCTAGCCTTGCTGGTGGTGCTGACTTCATGATGCGAGTCGTTGCCAAGAAGGTAACACTTAACGGAGCCACTACGACTTACCGAGAGTTGATTGGTGAGGAACAGTGGAACGCTCTCATGGTCACAGAACAACGTTCTCAGTTTGAGACTGACGCGAAGCTGAACGAGCAGTATCGCTTGGATATTAACTCTGCGCTGAACCAAGAGGACCCAAGGACAGCTTGGGAGATGCTTCAAGGTATCAAGGCTGAACTAGATAAGGTCCAACCTGATGAGCAGATGACACCACAACg                                                                                                                                                                                           <  1:656627/296‑1 (MQ=255)`  
                                                                                                               `ggACGCTTCTAGCCGTGCTGGTGGTGCTGACTTCCTGATGCGAGTCGGTGACAAGAAGGTAACACTTAACGGAGCCACTACGACTTACCGAGAGTTGATTGGTGAGGAACAGTGGAACGCTCTCATGGTCACAGCACAACGTTCTCAGTTTGAGACTGACGCGAAGCTGAACGAGCAGTATCGCTTGGAGATTAACTCTGCGCTGAACCAAGAGGACCCAAGGACAGCTTGGGAGATGCTTCAAGGTATCAAGGCTGAACTAGATAAGGTCCAACCTGATGAGCAGATGACACCACAACGt                                                                                                                                                                                          <  1:152747/300‑1 (MQ=255)`  
                                                                                                                    `cTTCTAGCCGTGCTGGTGGTGCTGACTTCCTGATGCGAGTCGGTGACAAGAAGGTAACACTTAACGGAGCCACTACGACTTCCCGAGAGTTGATTGGTGAGGAACAGTGGAACGCTCTCATGGTCACAGCACAACGTTCTCAGTTTGAGACTGACGCGAAGCTGAACGAGCCGTATCGCTTGGGGATTAACTCTGCGCTGAACCACGAGGACCCAAGGACAGCTTGGGAGATGCTTCAAGGTATCAAGGCTGAACTAGATAAGGTCCAACCTGATGAGctgtctcttatacacatctccga                                                                                                                                                                                     >  1:235900/1‑279 (MQ=255)`  
                                                                                                                    `cTTCTAGCCGTGCTGGTGGTGCTGACTTCCTGATGCGAGTCGGTGACAAGAAGGTAACACTTAACGGAGCCACTACGACTTACCGAGAGTTGATTGGTGAGGAACAGTGGAACGCTCTCATGGTCACAGCACAACGTTCTCAGTTTGAGACTGACGCGAAGCTGAACGAGCAGTATCGCTTGGAGATTAACTCTGCGCTGAACCAAGAGGACCCAAGGACAGCTTGGGAGATGCTTCAAGGTATCAAGGCTGAACTAGATAAGGTCCAACCTGATGAGCAGATGACACCACAACGTGAGTg                                                                                                                                                                                     >  1:326557/1‑301 (MQ=255)`  
                                                                                                                    `cTTCTAGCCGTGCTGGTGGTGCTGACTTCCTGATGCGAGTCGGTGACAAGAAGGTAACACTTAACGGAGCCACTACGACTTACCGAGAGTTGATTGGTGAGGAACAGTGGAACGCTCTCATGGTCACAGCACAACGTTCTCAGTTTGAGACTGACGCGAAGCTGAACGAGCAGTATCGCTTGGAGATTAACTCTGCGCTGAACCAAGAGGACCCAAGGACAGCTTGGGAGATGCTTCAAGGTATCAAGGCTGAACTAGATAAGGTCCAACCTGATGAGCAGATGACACCACAACGTGAGTg                                                                                                                                                                                     >  1:320525/1‑301 (MQ=255)`  
                                                                                                                          `gCCGTGCTGGTGGTGCTGACTTCCTGATGCGATTCGTTGACACGAATGTAACACTTAACGGAGCCACTACGACTTACCGAGCGATGATTGGTGAGGAACAGTGGAACACTCTCATGCTAACCGCACAACGTTCTCAGTTTGCGACTGACGCGATGCTGATCGAGTAATAACGCTTGGAGATCAACTCTGCGCTGAACCAAGAAGACCCAAGCCAAGCTCGGTAGATGCATCAAGTTATCAAGGATGAACTAGAGAAGGTCAAACATGATGAGCAGACGACACCACAACGGGCGTGGCTact                                                                                                                                                                               >  1:446666/1‑299 (MQ=255)`  
                                                                                                                           `aagaggtggaggagatGAATTACTGAGGCGAGTAGGAGAAAAGAAGGAAAAACATAACGGAGCAACAACAAAAAAACGAGAGTAGATTGGTGAGAAAAAGTGGAACGGTCTAAAGGACAAAGAACAACGTTATAAGTATGAGAGTGACGCGAAGATGAAAGAGTAGTAACGCATGGAGAGTAACAAAGCGCGGAAACAAGAGGACCCAAGGAAAGAGTGGGAGAAGAAGAAAGGAATCAAGGCTGAACTAGATAAGGTCCAACCTGATGAGCAGATGACACCACAACGTGAGTGGCTAAtc                                                                                                                                                                              <  1:279771/286‑1 (MQ=255)`  
                                                                                                                              `tGCTGGTGGTGCTGACTTCCTGATGCGAGTCGGTGACAAGAAGGTAACACTTAACGGAGCCACTACGACTTACCGAGAGTTGATTGGTGAGGAACAGTGGAACGCTCTCATGGTCACAGCACAACGTTCTCAGTTTGAGACTGACGCAAAGCTGAACGAGCAGTATCGCTTGGAGATTAACTCTGCGCTGAACCAAGAGGACCCAAGGACAGCTTGGGAGATGCTTCAAGGTATCAAGGCTGAACTAGATAAGGTCCAACCTGATGAGCAGATGACACCACAACGTGAATGGcctgtctct                                                                                                                                                                           >  1:518992/1‑293 (MQ=255)`  
                                                                                                                                `caggtggtGCTGACGTCCTGATGCGAGTCGGTGAGAAGAAGGTAACACTTAACGGAGCCACTACGACTTACCGAGAGTTGATTGGTGAGGAACAGTGGAACGCTCTCATGGTCACAGCACAACGTTCTCAGTTTGAGACTGACGCGAAGCTGAACGAGCAGTATCGCTTGGAGATTAACTCTGCGCTGAACCAAGAGGACCCAAGGACAGCTTGGGAGATGCTTCAAGGTATCAAGGCTGAACTAGATAAGGTCCAACCTGATGAGCAGATGACACCACAACGTGAGTGGCTAATCTCCGc                                                                                                                                                                         <  1:337841/299‑1 (MQ=255)`  
                                                                                                                                        `tcTGACTTCCTGATGCGAGTCGGTGACAAGAAGGTAACACTTAACGGTGCCACTACGACTTACCGAGAGTTTATTGGTGAGGAACAGTGGAACGCTCTCATGGTCACAGCACTACGTTCTCAGTTTGAGACTGACGCGAAGCTGAACGAGCAGTATCGCTTGGAGATTAACTCTGCGCTGAACCAAGAGGACCCAAGGACATCTTGGGAGATGCTTCAAGGTATCAAGGCTGAACTGGATAAGGTCCAACCTGATGAGCAGATGACACCACAACGTGAGTGGCTAATCTCCGCACAGGAAc                                                                                                                                                                 <  1:486219/300‑1 (MQ=255)`  
                                                                                                                                        `gCTGACTTCCTGATGCGAGTCGGTGACAAGAAGGTAACACTTAACGGAGCCACTACGACTTACCGAGAGTTGATTGGTGAGGAACAGTGGAACGCTCTCATGGTCACAGCACAACGTTCTCAGTTTGAGACTGACGCGAAGCTGAACGAGCAGTATCGCTTGGAGATTAACTCTGAGCTGAACCAAGAGGACCCAAGGACAGCTTGGGAGATGCTTCAAGGTATCAAGGCTGAACTAGATAAGGTCCAACCTGATGAGCAGAGGACACCACAACGTGAGTGGCTAATCTCCGCACAGGaaa                                                                                                                                                                 >  1:203180/1‑300 (MQ=255)`  
                                                                                                                                           `gACTTCCTGATGCGAGTCGGTGACAAGAAGGTAACACTTAACGGAGCCACTACGACTTACCGAGAGTTGATTGGTGAGGAACAGTGGAACGCTCTTATGGTCACAGCACAACGTTCTCAGTTTGAGACTGACGCGAAGCTGAACGAGCAGTATCGCTTGGAGATTAACTCTGCGCTGAACCAAGAGGACCCAAGGACAGCTTGGGAGATGCTTCAAGGTATCAAGGCTGAACTAGATAAGGTCCAACCTGATGAGCAGATGACACCACAACGTGAGTGGCTAATCTCCGcctgtctcttat                                                                                                                                                              >  1:544704/1‑290 (MQ=255)`  
                                                                                                                                            `atgtggataagagacagggTGACAAGGAGGTAACACTTAACGGAGCCACTACGACTTACCGAGAGTTGATTGGTGAGGAACAGTGGAACGCTCTCATGGTCACAGCACAACGTTCTCAGTTTGAGACTGACGCGAAGCTGAACGAGCAGTATCGCTTGGAGATTAACTCTGCGCTGAGCCAAGAGGACCCAAGGACAGCTTGGGAGATGCTTCAAGGTATCAAGGCTGAACTAGATAAGGTCCAACCTGATGAGCAGATGACACCACAACGTGAGTGGCTAATCTCCGCACAGGAACAAGt                                                                                                                                                             <  1:159910/284‑1 (MQ=255)`  
                                                                                                                                             `cTTCCTGATGCGAGTCGGTGACAAGAAGGTAACACTTAACGGAGCCACTACGACTTACCGAGAGTTGATTGGTGAGGAACAGTGGAACGCTCTCATGGTCACAGCACAACGTTCTCAGTTTGAGACTGACGCGAAGCTGAACGAGCAGTATCGCTTGGAGATTAACTCTGCGCTGAACCAAGAGGACCCAAGGACAGCTTGGGAGCTGCTTCAAGGTATCAAGGCTGAACTAGATAAGGTCCAACCTGATGAGCAGATGACACCACAACGTGAGTGGCTAATCTCCGCACAGGAACAAgct                                                                                                                                                            >  1:468364/1‑299 (MQ=255)`  
                                                                                                                                             `cTTCCTGATGCGAGTCGGTGACAAGAAGGTAACACTTAACGGAGCCACTACGACTTACCGAGAGTTGATTGGTGAGGAACAGTGGAACGCTCTCATGGTCACAGCACAACGTTCTCAGTTTGAGACTGACGCGAAGCTGAACGAGCAGTATCGCTTGGAGATTAACTCTGCGCTGAACCAAGAGGACCCAAGGACAGCTTGGGAGATGCTTCAAGGTATCAAGGCTGAACTAGATAAGGTCCAACCTGATGATCAGATGACACCACAACGTGAGTGGCTAATCTCCGCACAGGACCAAGtt                                                                                                                                                            >  1:431061/1‑301 (MQ=255)`  
                                                                                                                                                              `gTGACAAGAAGGTAACACTTAACGGAGCCACTACGCCTTACCGAGAGTTGATTGGTGAGGAACAGTGGAACGCTCTCATGGTCACAGCACAACGTTCTCAGTTTGCGACTGACGCGAAGCTGAACGAGCAGTATCGCTTGGAGATTAACTCTGCGCTGAACCAAGAGGACCCAAGGACAGCTTGGGAGATGCTTCAAGGTATCAAGGCTGAACTAGATAAGGTCCAACCTGATGAGCAGATGACACCACAACGTGAGTGGCTAATCTCCGCACAGGAACAAGTTCAGAATCAGATGAACGc                                                                                                                                           <  1:634394/301‑1 (MQ=255)`  
                                                                                                                                                                     `gaagGTAACACTTAACGGAGCCACTACGACTTACCTAGAGTTGATTGGTGAGGAACAGTGGAACGCTCTCATGGTCACAGCACAACGTTCTCAGTTTGAGACTGACGCGAAGCTGAACGAGCAGTATCGCTTGGAGATTAACTCTGCGCTGAACCAAGAGGACCCAAGGACAGCTTGGGAGATGCTTCAAGGTATCAAGGCTGAACTAGATAAGGTCCAACCTGATGAGCAGATGACACCACAACGTGAGTGGCTAATCTCCGCACAGGAACAAGTTCAGAATCAGATGAACGCATGGACg                                                                                                                                    >  1:667606/1‑301 (MQ=255)`  
                                                                                                                                                                          `aaaCACTTAACAGAGCCACTACGACTTACCGAGAGTTGATTGGTGAGGAACAGTGGAACGATCTCATGGTCACAGCACAACGTTCTCAGTTTGAGACTGACGCGAAGCTGAACGAGCAGTATCGCTTGGAGATTAACTCTGCGCTGAACCAAGAGGACCCAAGGACAGCTTGGGAGATGCTTCAAGGTATCAAGGCTGAACTAGATAAGGTCCAACCTGATGAGCAGATGACACCACAACGTGAGTGGCTAATCTCCGCACAGGAACAAGTTCAGAATCAGATGAACGCATGGACGAAAGc                                                                                                                               <  1:309077/300‑1 (MQ=255)`  
                                                                                                                                                                              `acTTAACGGAGCCACTACGACTTACCGAGAGTTGATTGGTGAGGAACAGTGGAACGCTCTCATGGTCACAGCACAACGTTCTCAGTTTGAGACTGACGCGAAGCTGAACGAGCAGTATCGCTTGGAGATTAACTCTGCGCTGAACCAAGAGGACCCAAGGACAGCTTGGGAGATGCTTCAAGGTATCAAGGCTGAACTAGATAAGGTCCAACCTGATGAGCAGATGACACCACAACGTGAGTGGCTAATCTCCGCACAGGAACAAGTTCAGAATCAGATGAACGCATGGACGAAAGCTCAg                                                                                                                           >  1:301832/1‑301 (MQ=255)`  
                                                                                                                                                                                 `tAACGGAGCCACTACGACTTACCGAGAGTTGATTGGTGAGGAACAGTGGAACGCTCTCATGGTCACAGCACAACGTTCTCAGTTTGAGACTGACGCGAAGCTGAACGAGCAGTATCGCTTGGAGATTAACTCTGCGCTGAACCAAGAGGACCCAAGGACAGCTTGGGAGATGCTTCAAGGTATCAAGGCTGAACTAGATAAGGTCCAACCTGATGAGCAGATGACACCACAACGTGAGTGGCTAATCTCCGCACAGGAACAAGTTCAGAATCAGATGAACGCATGGACGAAAGCTCAGGcc                                                                                                                        <  1:177878/301‑1 (MQ=255)`  
                                                                                                                                                                                          `cACTACGACTTACCGAGAGTTGATTGGTGAGGAACAGTGGAACGCTCTCATGGTCACAGCACAACGTTCTCAGTTTGAGACTGACGCGAAGCTGAACGAGCAGTATCGCTTGGAGATTAACTCTGCGCTGAACCAAGGGGACCCAAGGACAGCTTGGGAGATGCTTCAAGGTATCAAGGCTGAACTAGATAAGGTCCAACCTGATGAGCAGATGACACCACAACGTGAGTGGCTAATCTCCGCACAGGAACAAGTTCAGAATCAGATGAACGCATGGACGAAAGCTCAGGCCAAGGCTCTg                                                                                                               >  1:297056/1‑301 (MQ=255)`  
                                                                                                                                                                                                            `gTTGATTGGTGAGGAACAGTGGAACTCTCTCATGGTCACAGCACAACGTTCTCAGTTTGAGACTGACGCGAAGCTGAACGAGCAGTATCGCTTGGAGATTAACTCTGCGCTGAACCAAGAGGACCCAAGGACAGCTTGGGAGATGCTTCAAGGTATCAAGGCTGAACTAGATAAGGTCCAACCTGATGAGCAGATGACACCACAACGTGAGTGGCTAATCTCCGCACAGGAACAAGTTCGGAATCAGATGAACGCATGGACGAAAGCTCAGGCCAAGGCTCTGGACGATTCCATGAAGTCa                                                                                             >  1:213596/1‑301 (MQ=255)`  
                                                                                                                                                                                                                 `ttGGTGAGGAACAGTGGAACGCTCTCATGGTCACAGCACAACGTTCTCAGTTTGAGACTGACGCGAAGCTGAACGAGCAGTATCGCTTGGAGATTAACTCTGCGCTGAACCAAGAGGACCCAAGGACAGCTTGGGAGATGCTTCAAGGTATCAAGGCTGAACTAGGTAAGGTCCAACCTGATGAGCAGATGACACCACAACGTGAGTGGCTAATCTCCGCACAGGCACAAGTTCAGAATCAGATGAACGCATGGACGAAAGCTCAGGCCAAGGCTCTTGACGATTCCATGAAGTCAATGaa                                                                                        >  1:413713/1‑301 (MQ=255)`  
                                                                                                                                                                                                                   `ggTGAGGAACAGTGGAACGCTCTCATGGTCACAGCACAACGTTCTCAGTTTGAGACTGACGCGAAGCTGAACGAGCAGTATCGCTTGGAGATTAACTCTGCGCTGAACCAAGAGGACCCAAGGCCAGCTTGGGCGATGCTTCAAGGTATCAAGGCTGAACTAGATAAGGTCCAACCTGATGAGCAGATGACACCACAACGTGCGTGGCTAATCTCCGCACAGGAACAAGTTCAGAAACAGATGAAAGCATGGACGAAAGCTCAGGCCAATGCTCTAGACGCTTCCCTGAATGCAATGAACa                                                                                      >  1:42051/1‑301 (MQ=255)`  
                                                                                                                                                                                                                    `gTGAGGAACAGTGGAACGCTCTCATGGTCACAGCACAACGTTCTCAGTTTGAGACTGACGCGAAGCTGAACGAGCAGTATCGCTTGGAGATTAACTCTGCGCTGAACCAAGAGGACCCAAGGACAGCTTGGGAGATGCTTCAAGGTATCAAGGCTGAACTAGATAAGGTCCAACCTGATGAGCAGATGACACCACAACGTGAGTGGCTAATCTCCGCACAGGAACAAGTTCAGAATCAGATGAACGCATGGACGAAAGCTCAGGCCAAGGCTCTGGACGATTCCATGAAGTCAATGAACaa                                                                                     >  1:372154/1‑301 (MQ=255)`  
                                                                                                                                                                                                                         `gAACAGTGGAACGCTCTCATGGTCACAGCACAACGTTCTCAGTTTGAGACTGACGTGAAGCTGAACGAGCAGTATCGCTTGGAGATTAACTCTGCGCTGAACCAAGAGGACCCAAGGACAGCTTGGGAGATGCTTCAAGGTATCAAGGCTGAACTAGATAAGGTCCAACCTGATGAGCAGATGACACCACAACGTGAGTGGCTAATCTCCGCACAGGAACAAGTTCAGAATCAGATGAACGCATGGACGAAAGCTCAGGCCAAGGCTCTGGACGATTCCATGAAGTCAATGAActgtctct                                                                                >  1:224957/1‑294 (MQ=255)`  
                                                                                                                                                                                                                            `cAGTGGAACGCTCTCATGGTCACAGCACAACGTTCTCAGTTTGAGACTGACGCGAAGCTGAACGAGCAGTATCGCTTGGAGATTAACTCTGCGCTGAACCAAGAGGACCCAAGGACAGTTTGGGAGATGCTTCAAGGTATCAAGGCTGAACTAGATAAGGTCCAACCTGATGAGCAGATGACACCACAACGTGAGTGGCTAATCTCCGCACAGGAACAAGTTCAGAATCAGATGAACGCATGGACGAAAGCTCAGGCCAAGGCTCTGGACGATTCCATGACGTCAATGAACAAACtctgtc                                                                             >  1:74593/1‑296 (MQ=255)`  
                                                                                                                                                                                                                                  `aaCGCTCTCATGGTCACAGCACAACGTTCTCAGTTTGAGACTGACGCGAAGCTGAACGAGCAGTATCGCTTGGAGATTAACTCTGCGCTGAACCAAGAGGACCCAAGGACAGCTTGGGAGATGCTTCAAGGTATCAAGGCTGAACTAGATAAGGTCCAACCTGATGAGCAGATGACACCACAACGTGAGTGGCTAATCTCCGCACAGGAACAAGTTCAGAATCAGATGAACGCATGGACGAAAGCTCAGGCCAAGGCTCTGGACGATTCCATGAAGTCAATGAACAAACTTGACGTAATCg                                                                       <  1:218802/301‑1 (MQ=255)`  
                                                                                                                                                                                                                                   `aCGCTCTCATGGTCACAGCACAACGTTCTCAGTTTGAGACTGACGCGAAGCTGAACGAGCAGTATCGCTTTGAGATTAACTCTGCGCTGAACCAAGAGGACCCAAGGACAGCTTGGTAGATGCTTCAAGGTATCAAGGCTGAACTAGATAAGGTCCAACCTGATGAGCAGATGACACCACAACGTGAGTGGCTAATCTCCGCACAGGAACAAGTTCAGAATCAGATGAACGCATGGACGAAAGCTCAGGCCAAGGCTCTGGACGATTCCATGAAGTCAATGAACAAACTTGACGTAATCGa                                                                      >  1:643965/1‑301 (MQ=255)`  
                                                                                                                                                                                                                                        `ctcATGGTCACAGCACAACGTTCTCAGTTTGAGACTGACGCGAAGCTGAACGAGCAGTATCGCTTGGAGATTAACTCTGCGCTGAACCAAGAGGACCCAAGGACAGCTTGGGAGATGCTTCAAGGTATCAAGGCTGAACTAGATAAGGTCCAACATGATGAGCAGATGACACCACAACGTGAGTGGCTAATCTCCGCACAGGAACAAGTTCAGGATAAGATGAACGCATGGACGAAAGCTCAGGCAAAGGCTCTGGACGAGTCCATGACGTCAATGAACAAACTTGACGAAATATACAAGc                                                                 >  1:141044/1‑301 (MQ=255)`  
                                                                                                                                                                                                                                                  `caGCACAACGTTCTCAGTTTGAGACTGACGCGAAGCTGAACGAGCAGTATCGCTTGGAGATTAACTCTGCGCTGAACCAAGAGGACCCAAGGACAGCTTGGGAGATGCTTCAAGGTATCAAGGCTGAACTAGATAAGGTCCAACCTGATGAGCAGATGACACCACAACGTGAGTGGCTAATCTCCGCACAGGAACAAGTTCAGAATCAGATGAACGCATGGACGAAAGCTCAGGCCAAGGCTCTGGACGATTCCATGAAGTCAATGCACAAACTTGACGTAATCGACAAGCAATTCCAGaa                                                       >  1:146987/1‑301 (MQ=255)`  
                                                                                                                                                                                                                                                    `gCACAACGTTCTCAGTTTGAGACTGACGCGAAGCTGAACGAGCAGTATCTCTTGGAGATTACCTCTGCGCTGAACCAAGAGGACCCAAGGACAGCTTGGGAGATGCTTCAAGGTATCAAGGCTGAACTAGATAAGGTCCCACCTGATGAGCAGATGACACCACAACGTGAGTGGCTAATCTTCGCCCAGGAACAAGTTCAGAATCAGATGAACGCATGGACGAAAGCTCAGGCCAAGGCTCTGGACGATTCCATGAAGTCAATGAACAAACTTGACGTAATCGACAAGCAATTCCAGAAGc                                                     >  1:285770/1‑301 (MQ=255)`  
                                                                                                                                                                                                                                                          `cGTTCTCAGTTTGAGACTGACGCGAAGCTGAACGAGCAGTATCGCTTGGAGATTAACTCTGCGCTGAACCAAGAGGACCCAAGGACAGCTTGGGAGATGCTTCAAGGTATCAAGGCTGAACTAGATAAGGTCCAACCTGATGAGCAGATGACACAACAACGTGAGTGGCTAATCTCCGCACAGGAACAAGTTCAGAATCAGATGAACGCATGGACGAAAGCTCAGGCCAAGGCTCTGGACGATTCCATGAAGTCAATGAACAAACTTGACGTAATCGACCAGCAATTCCAGAAGCGAATCa                                               >  1:402359/1‑301 (MQ=255)`  
                                                                                                                                                                                                                                                              `ctcAGTTTGAGACTGACGCGAAGCTGAACGAGCAGTATCGCTTGGAGATTAACTCTGCGCTGAACCAAGAGGACCCAAGGACAGCTTGGGAGATGCTTCAAGGTAACAAGGCTGAACTAGATAAGGTCCAACCTGATGAGCAGATGACACCACAACGTGAGTGGCTAATCTCCGCACAGGAACAAGTTCAGAATCAGATGAACGCATGGACGAAAGCTCAGGTCAAGGCTCTGGACGATTCCATGAAGTCAATGAACAAACTTGACGTAATCGACAAGCAATTCCAGAAGCGAATAAACgg                                           <  1:111644/301‑1 (MQ=255)`  
                                                                                                                                                                                                                                                                  `gTTTGAGACTGACGCGAAGCTGAACGAGCAGTATCGCTTGGAGATTAACTCTGCGCTGAACCAAGAGGACCCAAGGACAGCTTGGGAGATGCTTCAAGGTATCAAGGCTGAACTAGATAAGGTCCAACCTGATGAGCAGATGACACCACAACGTGAGTGGCTAATCTCCGCACAGGAACAAGTTCAGAATCAGATGAACGCATGGACGAAAGCTCAGGCCAAGGCTCTGGACGATTACATGAAGTCAATGAACAAACTTGACGTAATCGACAAGCAATTCCAGAAGCGAATCAACTGtctc                                       >  1:556894/1‑298 (MQ=255)`  
                                                                                                                                                                                                                                                                                    `gCTGAACGAGCAGTATCGCTTGGAGATTAACTCTGCGCTGAACCAAGAGGACCCAAGGACAGCTTGGGAGATGCTTCAAGGTATCAAGGCTGAACTAGATAAGGTCCAACCTGATGAGCAGATGACACCACAACGTGAGTGGCTAATCTCCGCACAGGAACAAGTTCAGAATCAGATGAACGCATGGACGAAAGCTCAGGCCAAGGCTCTGGACGATTCCATGAAGTCAATGAACAAACTTGACGTAATCGACAAGCAATTCCAGAAGCGAATCAACGGTGAGTGGGTCTCATCGGAtttt                     >  1:273020/1‑301 (MQ=255)`  
                                                                                                                                                                                                                                                                                       `gAACGAGCAGTATCGCTTGGAGATTAACTCTGCGCTGAACCAAGAGGACCCAAGGACAGCTTGGGAGATGCTTCAAGGTATCAAGGCTGAACTAGATAAGGTCCAACCTGATGAGCAGATGACACCACAACGTGAGTGGCTAATCTCCGCACAGGAACAAGTTCAGAATCAGATGAACGCATGGACGAAAGCTCAGGCCAAGGATCTGGACGATTCCATGAAGTCAATGAACAAACTTGACGTAATCGACAAGCAATTCCAGAAGCGAATCAACGGTGAGTGGGTCTCAACGGATTTTAAg                  >  1:58216/1‑301 (MQ=255)`  
                                                                                                                                                                                                                                                                                         `cagAGCAGTATCGCTTGGAGATTAACTCTGCGCTGAACCAAGAGGACCCAAGGACAGCTTGGGAGATGCTTCAAGGTATCAAGGCTGAACTAGATAAGGTCCAACCTGATGAGTAGATGACACCACAACGTGAGTGGCTAATCTCCGCACAGGAACAAGTTCAGAATCAGATGAACGCATGGACGAAAGCTCAGGCCAAGGCTCTGGACGATTCCATGAAGTCAATGAACAAACTTGACGTAATCGACAAGCAATTCCAGAAGCGAATCAACGGTGAGTGGGTCTCAACGGATTTTAAGGa                <  1:37661/299‑1 (MQ=255)`  
                                                                                                                                                                                                                                                                                               `aGTATCGCTTGGAGATTAACTCTGCGCTGAACCAATAGGACCCAAGGACAGCTTGGGAGATGCTTCAAGGTATCAAGGCTGAACTAGATAAGGTCCAACCTGATGAGCAGATGACACCACAACGTGAGTGGCTAATCTCCGCACAGGAACAAGTTCAGAATCAGATGAACGCATGGACGAAAGCTCAGGCCAAGGCACTGGACGATTCCATGAAGTCAATGAACAAACTTGACGTAATCGACAAGCAATTCCAGAAGCGAATCAACGGTGAGTGGGTCTCAACGGATTTTAAGGATATGcc          <  1:60694/301‑1 (MQ=255)`  
                                                                                                                                                                                                                                                                                                `gTATCGCTTGGAGATTAACTCTGCGCTGAACCAAGAGGACCCAAGGACAGCTTGGGAGATGCTTCAAGGTATCAAGGCTGAACTAGATAAGGTCCAACCTGATGAGCAGATGACACCACAACGTGAGTGGCTAATCTCCGCACAAGAACAAGTTCAGAATCAGATGAACGCATGGACGAAAGCTCAGGCCAAGGCTCTGGACGATTCCATGAAGTCAATGAACAAACTTGACGTAATCGACAAGCAATTCCAGAAGCGAATCAACGGTGAGTGGGTCTCAACGGATTTTAAGGATATGccc         >  1:612733/1‑300 (MQ=255)`  

                                                                                                                                                                                                                                                                                                          `|`                                                                                                                                                                                                                                                                                                           
`GCGTCGTCCAGACTCTGCTGACTTCTTTGAGAAGTATATCGACAACGGTCTGGTTACTGGCGCAATCCCATCTGATGCTCAAGCCACACAGCTTATAAGCCAAGCGTTCAGTGACGCTTCTAGCCGTGCTGGTGGTGCTGACTTCCTGATGCGAGTCGGTGACAAGAAGGTAACACTTAACGGAGCCACTACGACTTACCGAGAGTTGATTGGTGAGGAACAGTGGAACGCTCTCATGGTCACAGCACAACGTTCTCAGTTTGAGACTGACGCGAAGCTGAACGAGCAGTATCGCTTGAAGATTAACTCTGCGCTGAACCAAGAGGACCCAAGGACAGCTTGGGAGATGCTTCAAGGTATCAAGGCTGAACTAGATAAGGTCCAACCTGATGAGCAGATGACACCACAACGTGAGTGGCTAATCTCCGCACAGGAACAAGTTCAGAATCAGATGAACGCATGGACGAAAGCTCAGGCCAAGGCTCTGGACGATTCCATGAAGTCAATGAACAAACTTGACGTAATCGACAAGCAATTCCAGAAGCGAATCAACGGTGAGTGGGTCTCAACGGATTTTAAGGATATGCCAGTCAACG  >  NC_001604.1/28960‑29555`

  

Alignment Legend

Aligned base mismatch/match (shaded by quality score): `ATCG/ATCG` `< 3 ≤` `ATCG/ATCG` `< 10 ≤` `ATCG/ATCG` `< 22 ≤` `ATCG/ATCG` `< 34 ≤` `ATCG/ATCG` `< 38 ≤` `ATCG/ATCG`

Unaligned base: `atcg`    Masked matching base: `atcg`    Alignment gap: `‑`    Deleted base: `‑`
